# Association between paraspinal muscle fat infiltration and regional kyphosis angle in thoracolumbar fracture patients: a retrospective study

**DOI:** 10.1038/s41598-024-53017-z

**Published:** 2024-01-29

**Authors:** Yitao Liao, Xiaofeng Liu, Taichuan Xu, Chao Li, Qingming Xiao, Xian Zhang

**Affiliations:** 1 Nanjing University of Chinese Medicine, Nanjing, 210023 China; 2 Department of Spine, Wuxi Affiliated Hospital of Nanjing University of Chinese Medicine, Wuxi, 214071 China

**Keywords:** Diseases, Medical research

## Abstract

This study aims to evaluate the impact of percutaneous pedicle screw fixation (PPSF) and open pedicle screw fixation (OPSF) on the postoperative paraspinal muscle fat infiltration (FI) rate in patients with thoracolumbar fractures through magnetic resonance imaging (MRI), and explore the association between paraspinal muscle FI rate and regional kyphosis angle. We retrospectively analyzed clinical data from 35 patients who underwent either PPSF or OPSF for thoracolumbar fractures, examining data at preoperative, 1-month postoperative, and 9-months postoperative time points, which included Visual Analog Scale (VAS), Oswestry Disability Index (ODI), and regional kyphosis angle. We obtained preoperative and 9-month postoperative paraspinal muscle FI rates using T2-weighted MRI images and ImageJ software. We analyzed the correlation of FI rates with VAS, ODI, as well as the correction loss percentage of regional kyphosis angle. The analysis revealed a positive correlation between postoperative FI rate increase and correction loss percentage of regional kyphosis angle (r = 0.696, *p* < 0.001). The increase in paraspinal muscle FI rate was positively correlated with 9-month postoperative ODI (r = 0.763, *p* < 0.001). These findings indicate that an increase in postoperative paraspinal muscle FI rate may result in more significant correction loss of regional kyphosis angle and can lead to increased functional impairment in patients.

## Introduction

Compared to open pedicle screw fixation (OPSF) surgery, minimally invasive percutaneous pedicle screw fixation (PPSF) surgery for treating thoracolumbar fractures has been extensively proven to offer benefits^1,2^. One of the most widely acknowledged benefits is the reduction in damage to the paraspinal muscles. Evidence in literature indicates that when compared to open methods, minimally invasive surgery causes less harm to the paraspinal muscles, which play a crucial role in maintaining spinal stability and functionality^3^.

Spinal kyphotic deformity is a common complication of thoracolumbar fractures and can significantly impact patients' daily lives^4^. The recognition of the role of paraspinal muscles in spinal stability and degenerative changes is growing, such as Zhang's^5^ discovery that paraspinal muscle degeneration predicts Proximal Junctional Kyphosis and Katzman's^6^ observations about the potential impact of changes in paraspinal muscle composition on kyphosis. Patients with thoracolumbar fractures exhibited more prominent preoperative sagittal plane deformities, they were consequently more inclined to experience postoperative sagittal plane changes^7^. Literature indicates that the percutaneous approach is associated with less Cobb angle correction loss compared to the open approach^8^. Research also indicates that low back pain (LBP) and disability are associated with atrophy of multifidus muscles in the lumbar spine and increased FI^9^.

However, despite the numerous studies conducted on the role of paraspinal muscles, there is a lack of research focused on examining the relationship between changes in the FI rate at the level of the fractured vertebra and the regional kyphosis angle in postoperative populations with thoracolumbar fractures. We compared paraspinal muscle FI rate, VAS, ODI, and regional kyphosis angle between OPSF and PPSF groups at various postoperative time points to assess the impact of different surgical approaches. Subsequently, correlation analysis was employed to determine the relationship between FI rate and patients' VAS, ODI, and regional kyphosis angle. Our focus is on understanding the role of paraspinal muscle FI rate in postoperative regional kyphosis angle maintenance and its impact on pain and functional impairment in thoracolumbar spine patients. Through these insights, we can provide more precise guidance for clinical surgical decisions and formulate more tailored postoperative rehabilitation plans for patients. This will ultimately result in a decrease in functional disabilities among patients and a significant improvement in their quality of life.

## Materials and methods

### Patients

Approval from the ethics committee of our local ethics committee (Ethics Committee of Wuxi Hospital of Traditional Chinese Medicine.) (Approval number: 2023LUN-002-01) was obtained for this study design. The present study was performed in accordance with the contemporary amendments of the Declaration of Helsinki and within an appropriate ethical framework. The need for written informed consent from patients was waived by the Ethics Committee of Wuxi Hospital of Traditional Chinese Medicine because this was a retrospective study. In this study, we conducted observations on 35 thoracolumbar fracture patients who underwent OPSF or PPSF surgery at our institution from October 2018 to June 2022. According to AO Spine classification the inclusion criteria for our study were patients with T11-L3 single-segment type A fractures who had undergone either OPSF surgery or PPSF surgery, were between the ages of 18 and 65, and had complete follow-up and imaging data at preoperative, postoperative 1 month, and postoperative 9 months. Patients with concomitant nerve injury, scoliosis, non-traumatic fractures, a previous history of thoracic or lumbar spine surgery, or muscle diseases were excluded from the study. Both OPSF and PPSF can be applicable to type A thoracolumbar fractures. The medical team in the ward held a meeting to discuss and determine the surgical approach for the patient.

### Surgical technique

OPSF: After successful anesthesia, the patient is placed in the prone position. We performed standard disinfection and draping. Next, we made an incision along the central midline of the spinous processes, dissecting the subcutaneous tissues. Following this, a vertical incision was made approximately 1.5 cm lateral to the spinous processes. Muscular dissection and detachment were carried out from the spinous process and lamina using bipolar electrocautery, allowing visualization of the lower lamina and the outer border of the facet joint. Subsequently, we placed pedicle screws on either side of the adjacent vertebrae and used a connecting rod to support and restore the position of the injured vertebra. Finally, we securely fastened the fixation nuts to complete the surgery.

PPSF: After successful anesthesia, the patient is placed in a prone position, and routine disinfection and draping are performed. Firstly, the C-arm machine is used to locate the pedicle root shadow of the adjacent two vertebral bodies of the injured vertebra. Then, approximately 2.5 cm incisions are made along the marked positions of the pedicle root shadows, and the skin and fascia are incised. The puncture needle is inserted into the pedicle roots of the adjacent two vertebral bodies, about 0.5–0.8 cm from the posterior edge of the vertebral body, and the guide wire is inserted. Confirmation of the correct position of the puncture needle and guide wire is achieved under fluoroscopy. After satisfactory placement of the guide wire, the pedicle root screws are threaded through the guide wire, and four screws are smoothly inserted into the pedicle roots. Then, two connecting rods are inserted from the incision from top to bottom, and the locking nuts are tightened.

### Imaging assessments

**Figure 1:**
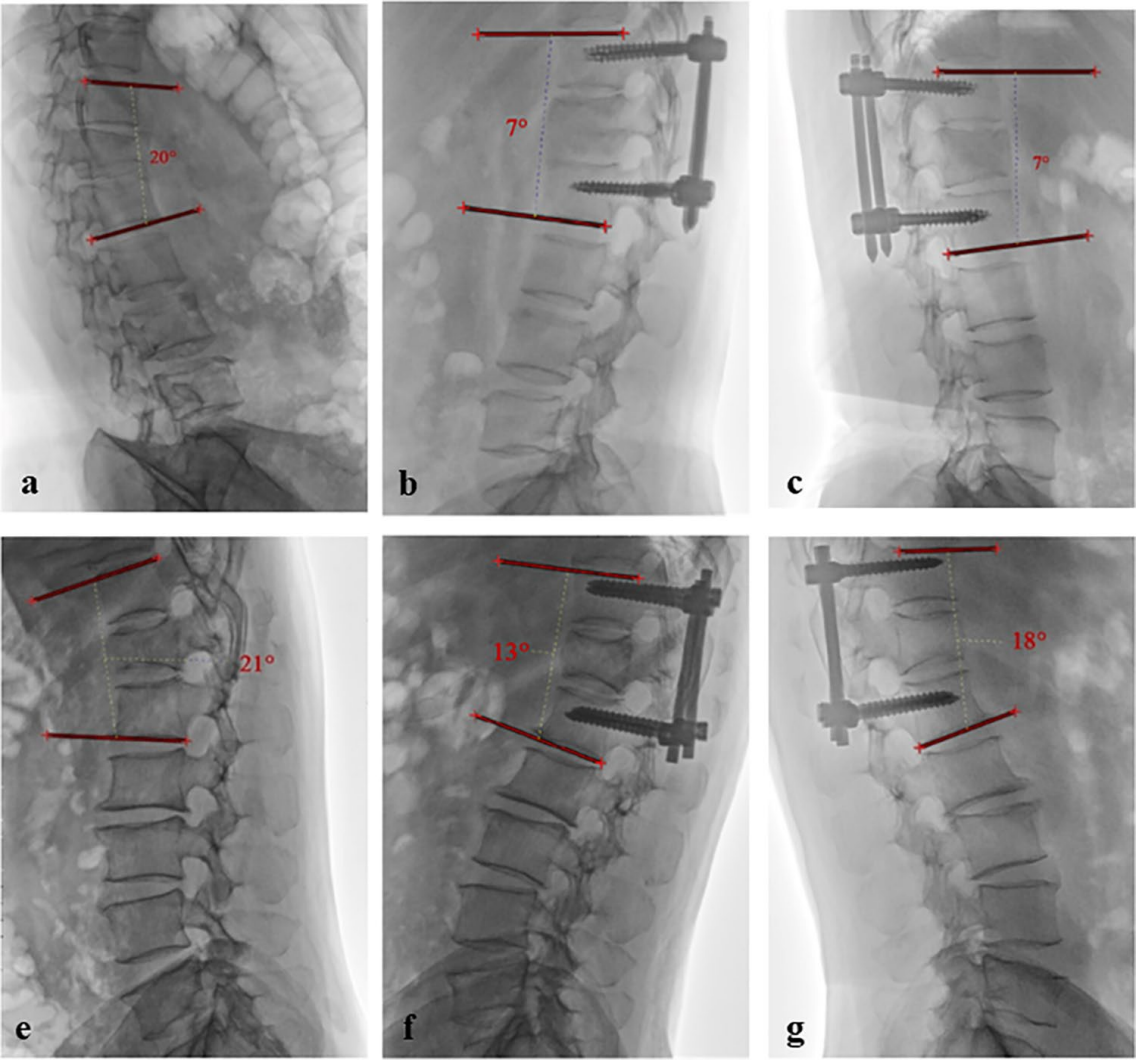
Regional kyphosis angle examples in two patient groups from different time periods. (**a**–**c**) represent the preoperative, 1-month postoperative, and 9-month postoperative angles for a patient in the PPSF group. The correction loss percentage of regional kyphosis angle for this patient was 0% [(7–7)/7*100%]. (**d**–**f**) depict the corresponding angles for a patient in the OPSF group. The correction loss percentage of regional kyphosis angle for this patient was 38.46% [(18–13)/13*100%].

**Figure 2:**
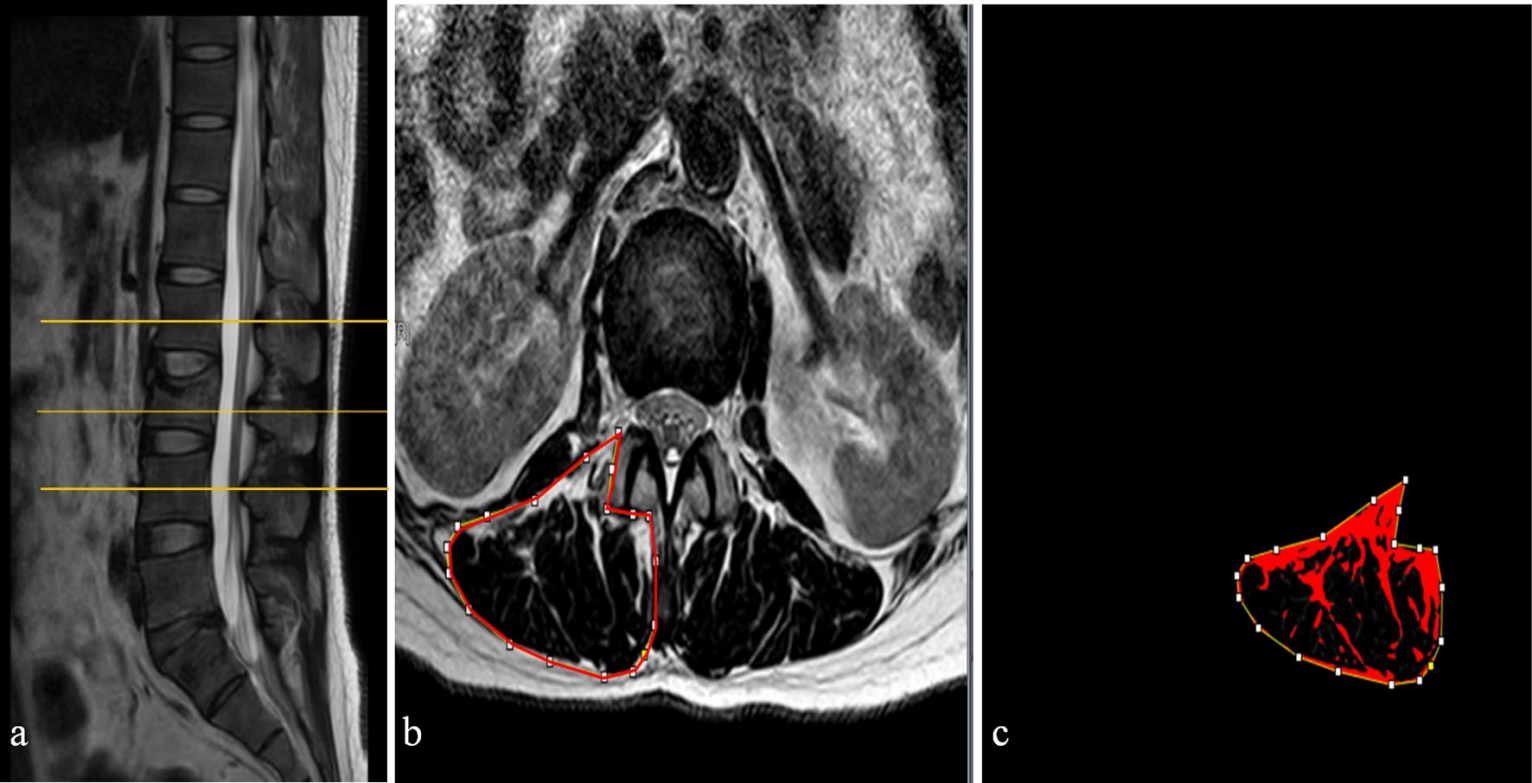
The patients' paraspinal muscle fat infiltration rates. The ROI for paraspinal muscles were drawn through the hospital's imaging system. The ROI fat infiltration rate was calculated using Image J software.

We collected preoperative, 1-month postoperatively, and 9-month postoperative lateral X-ray images from our hospital's imaging system, and measured their regional kyphosis angle. The regional kyphosis angle was determined by calculating the angle between the tangent to the upper endplate of the vertebra overlying the fracture and the tangent to the lower endplate of the vertebra underneath the injured vertebra. This measurement method has shown strong reliability and is endorsed by the Spine Trauma Group Study in the United States as a dependable approach^10,11^. To better represent the postoperative change in regional kyphosis angle, we calculated the correction loss percentage of regional kyphosis angle from 1 to 9 months postoperatively, i.e., (9-mo post angles—1-mo post angles)/1-mo angles × 100%. This is because the larger the preoperative regional kyphosis angle, the greater the regional kyphosis angle correction, and subsequently, a higher susceptibility to postoperative regional kyphosis angle loss^7^ (Fig. 1). We also collected preoperative and 9-month postoperative MRI (1.5-T Philips Achieva, The Netherlands, FOV: 180 mm, slice thickness: 4 mm, slice gap: 1 mm) images of the patients. Specifically, we focused on the fat infiltration rate of the paraspinal muscles, including the multifidus (MF) and erector spinae (ES) muscles. We measured the paraspinal muscle FI rate at the level of the injured vertebral body and the adjacent vertebral levels. The FI rate was measured on both sides, resulting in a total of six locations (Fig. 2a). T2-weighted cross-sectional MRI images were imported into Image J software, where the regions of interest (ROI) for paraspinal muscles were manually delineated (Fig. 2b). In this analysis, red areas represented fat tissue, and any pixel with a grey-scale value > 120 in Image J was considered as fat tissue in the paraspinal muscles^12–14^ (Fig. 2c). The fat infiltration rate is defined as the proportion of the red area to the ROI. The increase in FI rate from preoperative to 9 months postoperative was represented using Δ FI rate. To ensure measurement accuracy, we calculated the average values for these six locations. Two examiners independently assessed the regional kyphosis angle and fat infiltration rate. To determine the reliability of their observations, interclass correlation coefficients (ICC) were employed using a two-way mixed effects model.

### Clinical outcome assessments

We collected the patients' VAS and ODI scores preoperatively, at 1 month postoperatively, and at 9 months postoperatively.

### Statistical analysis

Data were analyzed using IBM SPSS (Version 25.0, SPSS, Inc., and Chicago, IL) statistical software. The normality of all data was assessed using the Kolmogorov–Smirnov test. Age, BMI, and VAS scores conformed to a normal distribution, while ODI and regional kyphosis angle exhibited distributions similar to normal. Descriptive statistics for these parameters were presented using mean and one standard deviation (SD). While for FI rate, median and interquartile range were utilized. We compared the OPSF and PPSF groups using the t-test for age and BMI, as well as Fisher's exact probability test for sex, fracture level, and fracture classification. We conducted a mixed ANOVA to assess differences in VAS, ODI, and the regional kyphosis angle between the OPSF and PPSF groups over time, followed by Bonferroni post-hoc tests for pairwise comparisons. Pairwise comparisons of the FI rates within each group before and after surgery were conducted using the Wilcoxon signed-rank test to assess differences. Differences in Δ FI rate and the correction loss percentage of the regional kyphosis angle between the two patient groups were evaluated using the Mann–Whitney U test. Use Spearman correlation coefficients to analyze the relationships between preoperative FI rate, 9-month post FI rate, and Δ FI rate with 9-month post VAS, ODI, and correction loss percentage of regional kyphosis angle for all patients. The Spearman correlation coefficient was understood as follows: less than 0.3 indicated no correlation; between 0.3 and 0.5 signified a weak correlation; a correlation between 0.5 and 0.7 was considered strong; 0.7 to 0.9 indicated a very strong correlation; and anything above 0.9 was deemed excellent. For all tests, *p* values < 0.05 were considered significant.

### Ethics statement

The studies involving human participants were reviewed and approved by the Ethics Committee of Wuxi Hospital of Traditional Chinese Medicine. This study has obtained informed consent from all participants.

## Results

### Patient characteristics

A total of 35 patients (13 male, 22 female; aged 31–65 years) were included, with 15 in the OPSF group and 20 in the PPSF group. Patient demographics are presented in Table 1.

**Table 1:** Patient demographics.

	OPSF	PPSF	*p*-value
Cases	15	20	
Age, years	54.05 ± 2.50	52.93 ± 1.50	0.351
Sex			1.000
Male	6	7	
Female	9	13	
BMI, m/kg^2^	23.93 ± 0.51	24.50 ± 0.54	0.549
Fracture level			1.000
T11	2	1	
T12	3	2	
L1	8	6	
L2	4	4	
L3	3	2	
Fracture classification			1.000
A0	0	0	
A1	7	9	
A2	2	3	
A3	5	8	
A4	1	0	

Values are the means ± SD (SD, standard deviation).

### Comparison of VAS

The analysis revealed a significant main effect of surgical type, *F* (1, 33) = 44.09, *p* < 0.001, partial *η*^2^ = 0.572, indicating differences in VAS scores between the two surgical procedures. Additionally, a significant main effect of assessment time was observed, with Greenhouse–Geisser adjusted *F* (1.43, 47.27) = 611.93, *p* < 0.001, partial *η*^2^ = 0.949, suggesting variations in VAS scores across different assessment times. The interaction effect between surgical type and time was not significant, as evidenced by Greenhouse–Geisser adjusted *F* (1.43, 47.27) = 1.70, *p* = 0.191, partial *η*^2^ = 0.049. Further scrutiny with Bonferroni multiple comparisons revealed that there were no significant differences in preoperative and 9-month postoperative VAS scores between the two patient groups. However, at 1 month postoperatively, the VAS scores in the PPSF group were significantly lower than those in the OPSF group, *p* < 0.001 (Table 2).

**Table 2:** Mixed ANOVA results for surgical impact on VAS, ODI, and regional kyphosis angle.

Variable	Time Point	OPSF	PPSF
VAS	Preoperative	8.67 ± 0.75	8.18 ± 0.89
	1-mo post	4.33 ± 0.62	3.32 ± 0.40
	9-mo post	2.47 ± 0.76	2.09 ± 0.55
ODI	Preoperative	82.00 ± 2.83	82.00 ± 3.24
	1-mo post	14.00 ± 2.00	8.80 ± 2.38
	9-mo post	6.80 ± 2.11	3.90 ± 1.20
Regional kyphosis angle	Preoperative	14.20 ± 5.43	14.40 ± 8.27
	1-mo post	5.27 ± 2.86	8.70 ± 4.73
	9-mo post	9.80 ± 5.24	8.90 ± 4.68

Values are the means ± SD (SD, standard deviation).

### Comparison of ODI

The analysis revealed a significant main effect of surgical procedure type,* F* (1, 33) = 22.08, *p* < 0.001, partial *η*^2^ = 0.401, the ODI scores of patients in the PPSF group were significantly lower than those in the OPSF group. Additionally, a significant main effect of assessment time was observed, with Sphericity Assumed *F* (2, 66) = 12,333.19, *p* < 0.001, and partial *η*^2^ = 0.997, suggesting variations in ODI scores across different assessment times. The interaction effect between surgical procedure type and time was found to be significant, as evidenced by Sphericity Assumed* F* (2, 66) = 11.53, *p* < 0.001, partial *η*^2^ = 0.259. This indicates a significant interaction effect between surgical procedure type and assessment time on ODI scores. The results of the simple effects tests indicated that there were no significant differences in patients' ODI scores between the two groups preoperatively. However, at 1-month and 9-month postoperative assessments, the ODI scores of patients in the PPSF group were significantly lower than those in the OPSF group, *p* < 0.001 (Table 2).

### Comparison of regional kyphosis angle

The main effect of group was not significant, *F* (1, 33) = 0.216, *p* = 0.645, partial *η*^2^ = 0.007, indicating no substantial differences in regional kyphosis angle between the OPSF and PPSF groups. However, a significant main effect of assessment time was observed, with Greenhouse–Geisser adjusted *F* (1.27, 41.89) = 44.04, *p* < 0.001, partial η^2^ = 0.572, suggesting notable variations in regional kyphosis angle across different assessment times. The interaction effect between surgical type and time was found to be significant, as evidenced by Greenhouse–Geisser adjusted *F* (1.27, 41.89) = 4.00, *p* = 0.023, partial *η*^2^ = 0.108. While the results of the simple effects tests indicated no significant differences in regional kyphosis angle between the two groups of patients at preoperative, 1-month postoperative, and 9-month postoperative assessments. Further scrutiny with Bonferroni multiple comparisons revealed that the regional kyphosis angle in both groups of patients at 1 month and 9 months after surgery was significantly smaller than the preoperative angle, *p* < 0.001. Additionally, in the PPSF group, there was no significant difference in the regional kyphosis angle between 9 months and 1 month after surgery. In contrast, in the OPSF group, the regional kyphosis angle at 9 months after surgery was significantly larger than at 1 month after surgery, *p* < 0.001 (Table 2). The OPSF group exhibits a significantly greater correction loss percentage of the regional kyphosis angle compared to the PPSF group, *p* < 0.001 (Table 3).

**Table 3:** Comparison of Δ FI rate and the correction loss percentage of regional kyphosis angle between the two patient groups.

	OPSF	PPSF	*p*-value
the correction loss percentage of regional kyphosis angle	26.67 (18.18, 42.86)	0 (0, 5.20)	< 0.001
Δ FI rate	11.19 (9.61, 14.84)	2.41 (0.83, 5.32)	< 0.001

The values are presented as the median and interquartile range.

### Comparison of FI rate

**Figure 3:**
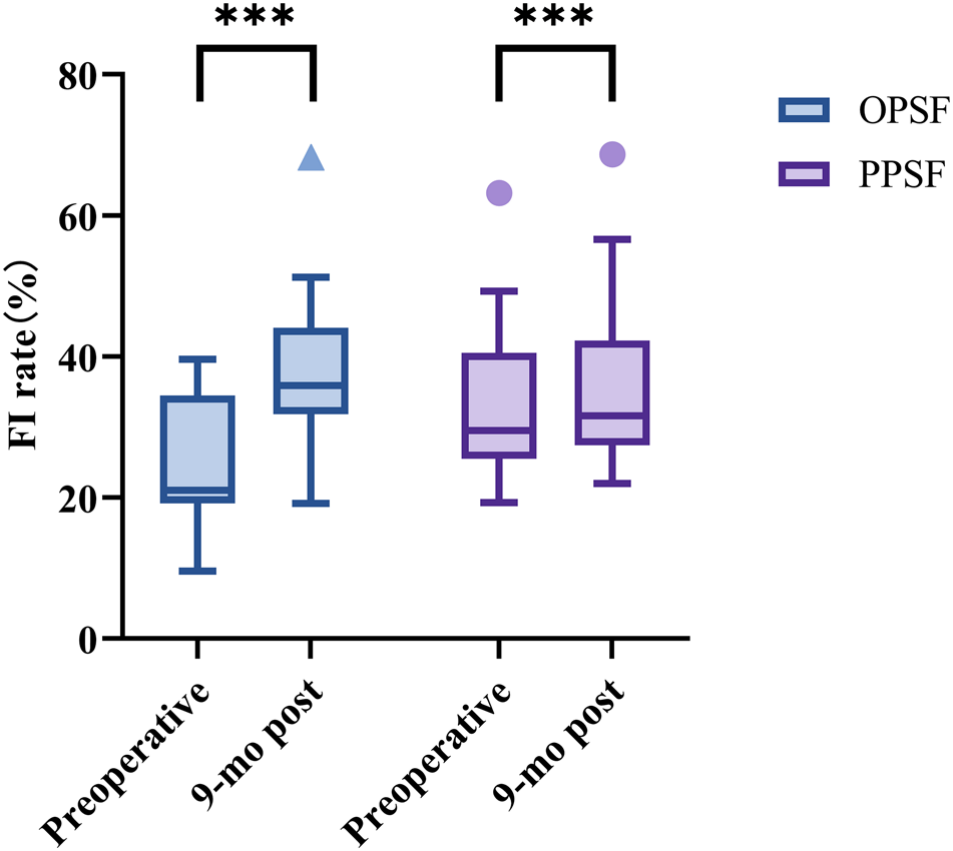
Comparison of FI rates within the same group preoperative and at 9 months postoperative. Both groups of patients had significantly higher FI rates at 9 months postoperatively compared to preoperative. The values are presented as the median and interquartile range (***p < 0.001).

Both groups of patients had significantly higher FI rates at 9 months postoperatively compared to preoperative (*p* < 0.001) (Fig. 3). However, the Δ FI rate in the OPSF group was significantly greater than that in the PPSF group (*p* < 0.001) (Table 3).

### Spearman correlation analysis

The results of the correlation analysis indicate that there was no significant correlation between preoperative FI rate and 9-mo post VAS. 9-mo post FI displayed a weak positive correlation with 9-mo post VAS (r = 0.367, *p* = 0.030), and Δ FI rate also exhibited a weak positive correlation with 9-mo post VAS (r = 0.335, *p* = 0.049). The correction loss percentage of regional kyphosis angle is negatively correlated with the preoperative paraspinal muscle FI rate (r = -0.349, *p* = 0.040), positively correlated with the Δ FI rate (r = 0.696, *p* < 0.001), and unrelated to the 9-mo post FI. The ODI scores at 9 months postoperatively showed no correlation with the preoperative FI rate or the FI rate at 9 months postoperatively. However, it does exhibit a positive correlation with the degree of increase in FI rate (r = 0.763, *p* < 0.001) (Table 4).

**Table 4:** Spearman correlation of the FI rate with 9-mo post VAS, 9-mo post ODI, and with the correction loss percentage of regional kyphosis angle.

		9-mo post VAS	9-mo post ODI	The correction loss percentage of regional kyphosis angle
Preoperative FI rate	r	0.091	− 0.277	− 0.349
	*p*-value	0.640	0.107	**0.040**
9-mo post FI rate	r	0.367	0.267	0.107
	*p*-value	**0.030**	0.121	0.540
Δ FI rate	r	0.335	0.763	0.696
	*p*-value	**0.049**	** < 0.001**	** < 0.001**

Significant values/results are printed in bold.

### ICC results

Inter-observer reliability showed excellent agreement, ICC (95% confidence interval)—the regional kyphosis angle: 0.984 (0.976, 0.989) and FI rate: 0.993 (0.988, 0.995).

## Discussion

A plethora of studies suggests that traditional open surgery is more likely to induce paraspinal muscle degeneration and atrophy. Tsutsumimoto et al.^15^ propose that in traditional open surgery, continuous traction on the paraspinal muscles raises the muscle pressure and impacts blood perfusion in paraspinal muscle capillaries. Yamamoto et al. 's^16^ study discovered that rats in the open surgical group, as compared to the sham surgery group, exhibited a reduced density of blood vessel distribution in the paraspinal muscles. This decrease in blood vessel density contributes to ischemic changes that exacerbate paraspinal muscle atrophy and degeneration. In the sham group, an incision was made in the skin and fascia lumbodorsalis at the midline. In the surgical group, paraspinal muscles were completely detached from the vertebrae, following the standard surgical procedure under anesthesia. Hu^17^ found that the splitting approach in open surgery remains a significant factor leading to multifidus muscle injury and atrophy during surgeries involving the posterior lumbar spine. Denervation and disuse may be important factors in multifidus muscle atrophy in the splitting approach, and this situation is more pronounced in conventional posterior open surgery. Compared with minimally invasive surgery, open surgery also leads to more severe paraspinal muscle edema, inflammation, and necrosis^9,18^. This provides a good basis for the formation of adipose tissue^17^. In open surgeries, the more frequent and extensive use of cautery devices may increase the likelihood of damaging the posterior branches of spinal nerves, thereby exacerbating paraspinal muscle atrophy^19^. Our results also showed a significantly smaller increase in the rate of paraspinal muscle FI in the PPSF group than in the OPSF group nine months after the procedure.

The relationship between paraspinal muscle FI and LBP is currently a subject of controversy. Many studies have indicated an association between an increase in the rate of paraspinal muscle FI and the occurrence of LBP^20,21^. Nevertheless, numerous studies have challenged the belief that an association exists between an increase in paraspinal muscle FI and LBP^22,23^. The correlation analysis results reveal a weak positive correlation between the postoperative 9-month paraspinal muscle FI rate and the postoperative VAS score. Additionally, Δ FI rate also exhibits a weak positive correlation with the postoperative 9-month VAS score. Despite these findings, it's crucial to recognize that, due to the inherent limitations of retrospective research, we cannot definitively conclude that an increase in paraspinal muscle fat infiltration directly leads to a higher likelihood of developing LBP. In light of this, it's essential to consider the broader context of factors influencing lower back pain. Hiyama et al.^24^ conducted a comparative investigation, examining short-term clinical outcomes in patients with degenerative spondylolisthesis and stenosis, focusing on the differences between extreme lateral interbody fusion and minimally invasive surgery-transforaminal interbody fusion. Their research suggests that minimally invasive surgery-transforaminal interbody fusion may lead to a more substantial improvement in lower back pain compared to extreme lateral interbody fusion. This difference in outcomes could be linked to variations in the invasiveness of the procedures, particularly their impact on posterior support elements such as facet joints, lamina, and paraspinal muscles. However, it's important to emphasize that the causes of LBP are multifaceted and diverse^24^. Factors such as patients' psychological backgrounds, spinal alignment, and medical histories can introduce significant biases and influence the development of LBP. Therefore, a comprehensive understanding of LBP must consider these complex interactions and individual variations.

The MF and ES muscles play essential roles in maintaining both the dynamic and static balance of the spine by providing strength and stability^25^. In their study on how skeletal muscle mass affects spine alignment in patients with spinal degenerative disease, Hiyama et al. found that skeletal muscle mass could be a significant factor influencing the posterior inclination of the pelvis in symptomatic spinal patients^26^. However, surgical procedures can cause fibrotic changes in the paraspinal muscles, leading to muscle stiffness and biomechanical alterations^16^. After surgery, there is a projected decrease in trunk extension strength by approximately 23%. Additionally, there is a reduction in multifidus fascicle force ranging from approximately 21% to 40% during flexion tasks and approximately 14% to 35% during upright tasks^27^. Biomechanical modeling further suggests that heightened muscle stiffness could lead to a more than 500% increase in compressive loads on the spine, thereby increasing the likelihood of spinal deformities^28^. Engaging in exercises targeting the paraspinal muscles can aid in slowing down the natural progression of spinal kyphosis^29^. All these pieces of evidence collectively emphasize the significance of paraspinal muscles in maintaining the sagittal balance of the spine. Furthermore, evidence indicates that it is possible to predict postoperative clinical outcomes and complications based on the preoperative morphology of the paraspinal muscles^30–32^.Our research findings similarly demonstrate a negative correlation between the preoperative FI rate and the correction loss percentage of regional kyphosis angle for the patients. We observed a positive correlation between the increase in the ΔFI rate and the correction loss percentage of regional kyphosis angle. However, no correlation was observed between the postoperative 9-month FI rate and the correction loss percentage of regional kyphosis angle. The correlation analysis between the FI rate and the postoperative 9-month ODI score yielded the same results. This is a thought-provoking issue. It seems that, in comparison to the postoperative paraspinal muscle FI rate alone, the extent of increase in postoperative FI rate may have a more significant impact on both the regional kyphosis angle and ODI score. Furthermore, these findings underscore the importance of protecting paraspinal muscles during surgery and minimizing damage to paraspinal muscles and tissues whenever possible. While this study solely observed changes in paraspinal muscle FI rate from preoperative to 9 months postoperative, existing literature indicates that the impact of surgical trauma to the paraspinal muscles persists. For instance, in a three-year patient follow-up, Lv et al.^33^ discovered that minimally invasive transforaminal lumbar interbody fusion provides benefits in preventing paraspinal muscle atrophy when compared to conventional transforaminal lumbar interbody fusion. It can be expected that this situation is even more pronounced in surgeries for patients with greater surgical trauma, as the removal of internal fixation devices can cause secondary harm to paraspinal muscles. Therefore, for patients undergoing surgeries with higher trauma, enhancing patient education is essential. Providing guidance on appropriate exercises targeting the paraspinal muscles, such as supine pelvic tilts to promote lumbar flexion/extension and supine bridging, can significantly contribute to the improvement of postoperative pain and functional impairments^34^. Therefore, when a posterior approach is not required for decompression or repositioning, we can consider an anterior approach surgery^35^.

The study has several noteworthy limitations. Firstly, the method used for assessing FI in this study, using T2 images, the accuracy is relatively low, with potential measurement errors arising from tissue edema or inflammation. A more precise approach, such as Chemical-shift MRI, which can generate water-only and fat-only images from dual-echo and/or multi-echo acquisitions, allowing for the quantification of both extramyocellular lipids and intramyocellular lipids^36^, should be considered for future investigations. Secondly, the study's relatively small sample size could potentially affect the reliability of our findings. Third, the observational period was limited to 9 months postoperatively. A longer follow-up would provide a more comprehensive understanding of the effects on sagittal spinal balance. It can be anticipated that after the removal of internal fixation, the role of paraspinal muscles in stabilizing the spine becomes more prominent. Therefore, based on this study, our research team will continue to investigate the long-term effects of postoperative paraspinal muscle degeneration on spinal stability. Furthermore, due to the retrospective design, only the regional kyphosis angle was assessed, which might have some impact on the evaluation of sagittal spinal balance. It's essential to recognize that while correlations have been identified, a definitive cause-and-effect relationship has not been firmly established. Our research team plans to design prospective studies building upon this research to further investigate the role of paravertebral muscles in maintaining spinal balance.

## Conclusion

In this retrospective study, we investigated the impact of different surgical approaches on paravertebral muscle FI rate in patients. Compared to OPSF, PPSF causes less damage to the paravertebral muscles in patients, and there is a smaller increase in the paravertebral muscle FI rate after surgery in the PPSF group. In the PPSF group, the correction loss percentage of the regional kyphosis angle is smaller. The correlation results show that the more the paravertebral muscle FI rate increases after surgery, the greater the correction loss percentage of the regional kyphosis angle, and the higher the VAS and ODI scores of the patients. Although a definite causal relationship has not been elucidated, our study suggests that paraspinal muscles play a crucial role in maintaining the correction of the regional kyphosis angle.

## Data Availability

Raw data will be made available on reasonable request and with the permission of the institution where the data were generated. Yitao Liao was the person to be contacted if someone wanted to request the data from this study.
